# Supplementary figures and images for: Self-Emulsifying Drug Delivery Systems: An Alternative Approach to Improve Brain Bioavailability of Poorly Water-Soluble Drugs through Intranasal Administration
Source: Pharmaceutics. 2022 Jul 18;14(7):1487. doi: 10.3390/pharmaceutics14071487 (PMC9319231; doi:10.3390/pharmaceutics14071487)

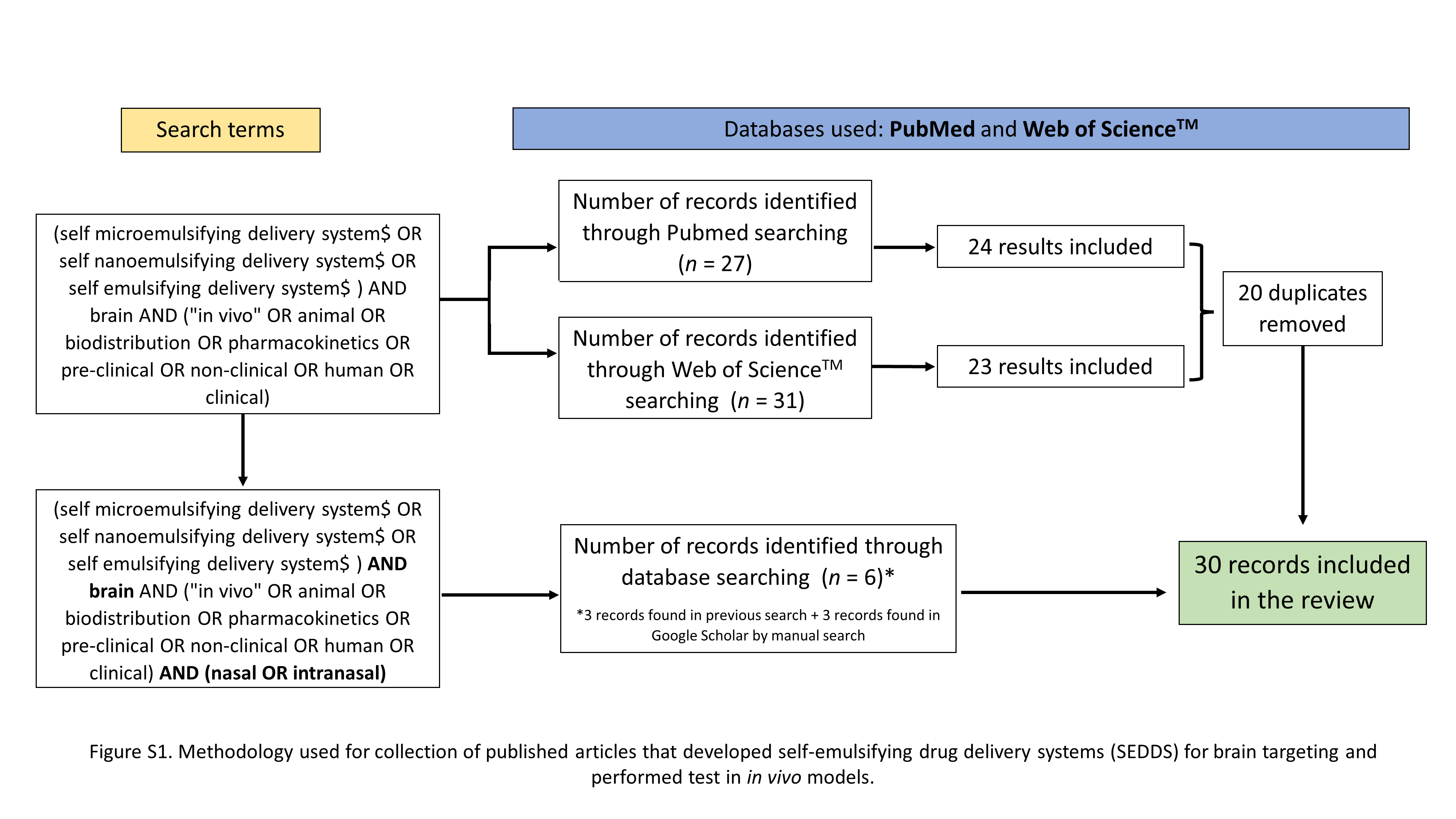

Supplement: Supplementary file 1 [file pharmaceutics-14-01487-s001.zip › pharmaceutics-1800591-supplementary.tif]
